# Respiratory anatomy and physiology in diving penguins

**DOI:** 10.1098/rstb.2023.0422

**Published:** 2025-02-27

**Authors:** P. J. Ponganis, C. L. Williams, M. Scadeng

**Affiliations:** ^1^ Scripps Institution of Oceanography, University of California San Diego, La Jolla, CA 92093, USA; ^2^ National Marine Mammal Foundation, 2240 Shelter Island Drive, San Diego, CA 92106, USA; ^3^ Department of Anatomy and Medical Imaging, Faculty of Health and Medical Sciences, University of Auckland, Auckland 1142, New Zealand; ^4^ Center for Functional Magnetic Resonance Imaging, University of California, San Diego, La Jolla, CA 92093, USA

**Keywords:** air sac, barotrauma, gas exchange, lung, oxygen store

## Abstract

The anatomy and function of the respiratory systems of penguins are reviewed in relation to gas exchange and minimization of the risks of pulmonary barotrauma, decompression sickness and nitrogen narcosis during dives. Topics include available lung morphology and morphometry, respiratory air volumes determined with different techniques, review of possible physiological and biomechanical mechanisms of baroprotection, calculations of baroprotection limits and review of air sac and arterial partial pressure of oxygen (P_O2_) profiles in relation to movement of air during breathing and during dives. Limits for baroprotection to 200, 400 and 600 m in Adélie, king and emperor penguins, respectively, would require complete transfer of air sac air and reductions in the combined tracheobronchial tree—parabronchial volume of 24% in Adélie, 53% in king penguins and 76% in emperor penguins. Air sac and arterial P_O2_ profiles at rest and during surface activity were consistent with unidirectional air flow through the lungs. During dives, P_O2_ profiles were more complex, but were consistent with compression of air sac air into the parabronchi and air capillaries with or without additional air mixing induced by potential differential air sac pressures generated by wing movements.

This article is part of the theme issue ‘The biology of the avian respiratory system’.

## 1. Introduction

In this review, we consider the anatomy and function of the respiratory systems of penguins in relation to gas exchange during dives and minimization of the risks of pulmonary barotrauma, decompression sickness and nitrogen narcosis. Importantly, gas exchange during a dive contrasts with that during flight and terrestrial exercise of most birds in that blood oxygen (O_2_) uptake and transport occur during a breath hold in a diving penguin. Increased respiratory rates are not an option to move air through the lungs and air sacs during a dive. Blood O_2_ uptake from the lungs during a dive is important, as the O_2_ in the air sacs and lungs (respiratory O_2_ store) is a significant portion (33–48%) of the total body O_2_ store (respiratory system, blood and muscle) in penguins [1]. In addition, because the air sacs of birds are about 10× larger than the lungs [2,3], movement of air into the lungs during a dive is necessary to access that O_2_.

Demands on the respiratory system during dives are best understood with consideration of dive performance. Common dives in most penguin species are typically 1–2 min in duration and less than 40 m in depth [1]. Little blue (*Eudyptula minor*) and Humboldt (*Spheniscus humboldti*) penguins, for example, usually dive for less than 2 min to depths of less than 10–20 m [4,5]. Deeper, longer dives occur routinely in three species (gentoo (*Pygoselis papua*): 80−100 m and 3−4 min, king (*Aptenodytes patagonicus*): 100−250 m and 2−6 min, and emperor (*Aptenodytes forsteri*): 100−500 m and 5−10 min) [6–9]. The effects of pressure and prolonged gas exchange at depth and the subsequent risks for barotrauma, decompression sickness and nitrogen narcosis are greatest in the deeper divers, especially king and emperor penguins. However, most shallow-diving species also face some risk as almost all of the shallow divers have occasional dives to 100–200 m depth [1].

**Figure 1.**
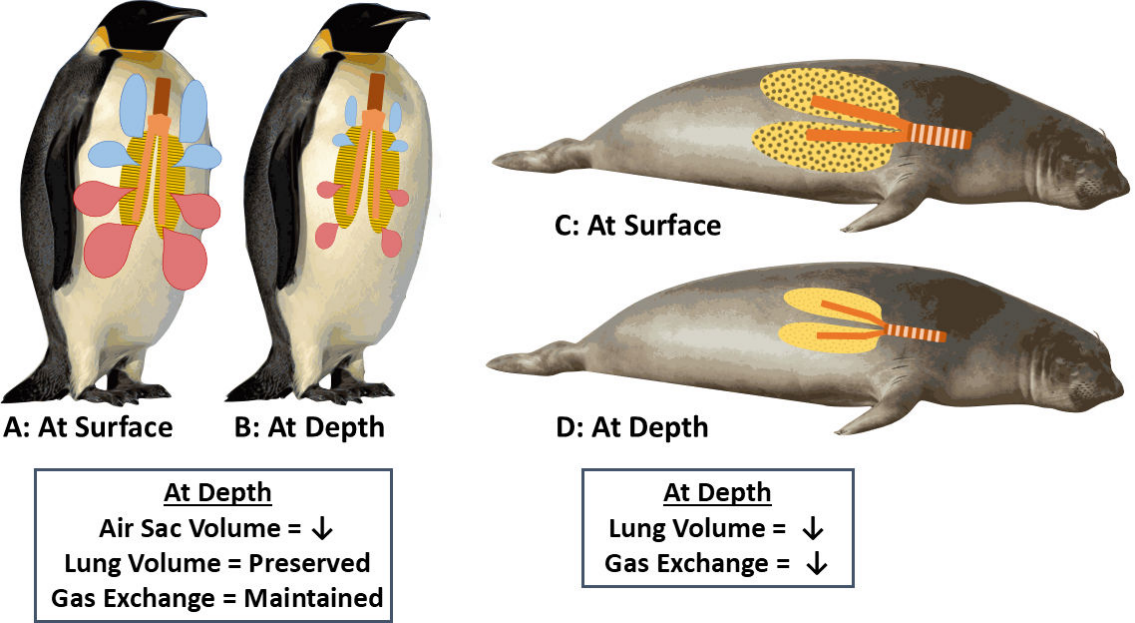
The response of the respiratory system to increased hydrostatic pressure differs between mammals and birds. In penguins, the lungs and tracheobronchial tree are less compliant (compressible) than the air sacs. By contrast, in mammals, the tracheobronchial tree is less compliant than the lungs. Consequently, in penguins, compression of the air sacs at depth results in a decrease in air sac volume and a transfer of air into the lungs with preservation of lung volume and gas exchange (*a* and *b*). In marine mammals, such as the seal, the alveoli of the lung are compressed, resulting in decreased lung volume and gas exchange as well as a transfer of air into the tracheobronchial tree (*c* and *d*).

We also remind readers here that the response of the respiratory system to increased hydrostatic pressure differs between mammals and birds (figure 1). When marine mammals dive to depth, the increased hydrostatic pressure compresses the respiratory system components, starting with the most compliant: the alveoli of the lung. As the alveoli are compressed, the air in the alveoli is forced into the less compliant tracheobronchial tree [10,11]. In marine mammals such as seals, complete alveolar collapse and cessation of gas exchange can occur at depth, minimizing the risks of decompression sickness and nitrogen narcosis [11–14]. By contrast, in the avian respiratory system, the lungs have long been considered immobile or ‘rigid’ (incompressible). In particular, the narrow air capillaries, the site of gas exchange in the lungs, have been thought unlikely to collapse during dives, even deep dives. However, the air sacs are highly compliant. Consequently, in penguins (figure 1), compression of the air sacs transfers air into the lungs where gas exchange can continue [15–17]. The continued gas exchange at depth can increase the risk of decompression sickness and nitrogen narcosis, yet deep-diving birds do not appear to suffer from these conditions after dives. The mechanisms that minimize excess nitrogen absorption and the risks of decompression sickness and nitrogen narcosis in penguins are still unknown.

With this brief introduction, we will review (i) respiratory air volumes, lung anatomy and baroprotection, (ii) risks of decompression sickness and nitrogen narcosis, (iii) air movement between the air sacs and lungs during breathing and during dives, and (iv) air sac and arterial partial pressure of O_2_ (P_O2_) at rest, and prior to and during dives with implications for gas exchange and intrapulmonary shunts.

## 2. Respiratory air volumes, lung anatomy and pulmonary baroprotection

### (a) Respiratory volumes

Air within the respiratory system of penguins and other birds is located in the lungs, air sacs and tracheobronchial tree. The lung is composed of lung tissue (structural tissue and blood within the pulmonary vasculature) and the air within the parabronchi and air capillaries. To understand the distribution of air within the respiratory system of penguins, we first review the size of the lungs, the morphology (form and structure) and morphometry (measurement of structural dimensions) of the lung, the volume of air (parabronchi and air capillaries) within the lung, the air volume of the air sacs and tracheobronchial volume. We then consider how these factors contribute to baroprotection and to gas exchange in the diving penguin.

#### (i) Lung volumes and morphometry

**Figure 2.**
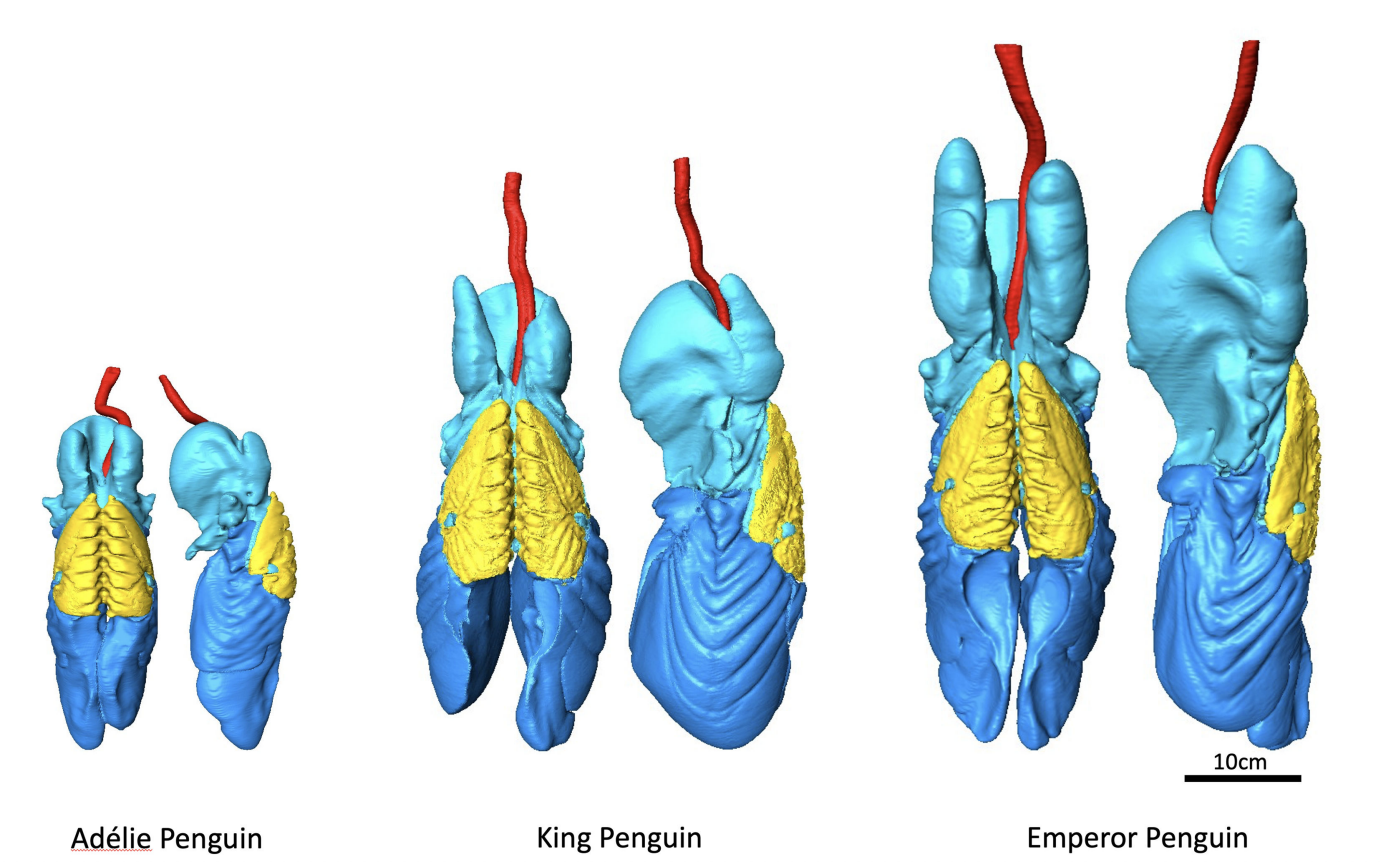
The respiratory systems of Adélie, king and emperor penguins, reconstructed from whole body serial CT scans of living birds, and scaled to body size. Colour code: trachea—red, anterior air sacs—light blue, posterior air sacs—blue, lungs—yellow. From the data of Ponganis [21].

The lung volumes (air+lung tissue) of Humboldt, Adélie (*Pygoscelis adeliae*), king and emperor penguins scaled allometrically with those of other birds [18–21]. The resulting mass specific lung volumes were lower in larger, deeper-diving species: Humboldt—30 ml kg^−1^, Adélie—25 ml kg^−1^, king—19 ml kg^−1^, emperor—18 ml kg^−1^ (table 1). These findings are consistent with the allometry of avian resting metabolic rate (log lung volume = 1.42877 + 0.96172 log body mass) [23,24]. In addition to size, the morphology and morphometry of the penguin lung is especially relevant to the air volume in the parabronchi and air capillaries. These air volumes are critical to the size of the respiratory O_2_ store and to the prevention of barotrauma. However, lung morphometry has only been investigated in one species, the shallow-diving Humboldt penguin [22]. Lung morphometry of the deeper diving species is needed, but we are aware of only one other penguin lung study, an ultrastructural study that found a thickened blood–gas barrier in emperor penguins [25]. Notably, in the Humboldt penguin, the blood–gas barrier was also thickened (3.4× greater than in several flighted avian species) and the mass specific surface area of the barrier was the lowest of the birds examined [22]. Together, these two aspects of the blood–gas barrier resulted in a mass specific anatomical diffusing capacity for oxygen that was only 15% the mean value of the flighted birds in the study. Total air volume in the Humboldt penguin lung was calculated as 58.9% of total lung volume; the primary bronchi (within the lung), parabronchi and air capillary volumes constituted 3.56%, 37.71% and 17.67% of total lung volume [19,22]. Therefore, these percentages were used to calculate component air volumes in Adélie, king and emperor penguins based on three-dimensional lung reconstructions from serial computerized tomographic (CT) scans [21] (table 1, figure 2).

**Table 1.** (*a*) Respiratory volumes and body masses in four penguin species. Adapted from Maina and King [22], Maina and Nathaniel [19] and Ponganis *et al.* [21]. Data for Adélie, king and emperor penguins are from table 2 of Ponganis *et al*. [21]. Abbreviation: TB, tracheobronchial. (*b*) Calculation of the percentage reduction of the combined total tracheobronchial tree-parabronchial space (TBT+PB) required to provide baroprotection for the deepest dives reported for Adélie, king and emperor penguins (see text). Starting at one atmosphere absolute (ATA) at the surface, ambient pressure increases 1 ATA every 10 m in depth, resulting in the volume ratios in the table. To avoid barotrauma, the total air volume at the surface (total air) divided by (TBT+PB + air capillary (AC) space)_at the threshold depth_ must equal the ratio for the threshold depth. These calculations assume highly compliant air sacs, an incompressible space for the air capillaries and a fractional reduction (*x*) in the tracheobronchial tree and parabronchial spaces secondary to smooth muscle constriction, blood engorgement and/or compression of these structures. The formula utilizes data in table 1*a*.

(*a*) species	Humboldt	Adélie	king	emperor
body mass (kg)	4.50	4.63	13.43	21.46
air + tissue volumes:				
total lung volume (ml kg^−1^)	30	25	19	18
total lung volume (ml)	135	115	252	353
air volumes (ml):				
lung air (total)	79	70	153	234
air capillaries	24	21	46	70
parabronchi	50	45	98	150
primary bronchi	5	4	9	14
extrapulmonary trachea and bronchi	NA	14	53	78
total TB tree (TBT)	NA	18	62	92
extrapulmonary trachea and bronchi + lung	NA	84	206	312
TBT + parabronchi	NA	63	160	242
air capillaries + parabronchi	74	66	144	220
air sac volume	NA	1356	4768	7482
total air	NA	1440	4974	7794

(*b*) species	barotrauma threshold depth (m)	required volume ratio for threshold depth	fraction (x) in ratio = total air/(x(TBT+PB)+AC)	% reduction for threshold depth (1−x)100
Adélie	200	21	0.755	24
king	400	41	0.471	53
emperor	600	61	0.239	76

#### (ii) Air sac volumes

By contrast to the relatively rigid avian lung, the high compliance of avian air sacs has long complicated measurements of air sac volume and total respiratory air volume. Air sac volume changes with depth of inspiration, body position, anaesthesia and spontaneous versus artificial ventilation [3,26,27]. Measurements determined with injection casts are dependent on injection/inflation pressure as well as cast material shrinkage/expansion [28,29]. Allometric analyses of the volume of the air sacs and of the entire respiratory system in birds have utilized measurements that were usually determined with injection casts [2,18]. Although such cast measurements have been considered maximal volumes, it is notable that inert gas techniques in the duck at rest have yielded total respiratory volumes that were 35% greater than predicted allometrically [3], and in the free-diving lesser scaup, air sac volumes were 2× the predicted value [30]. Consequently, the value of allometric predictions for air sac volume in a penguin at rest or prior to a dive is unresolved.

Scholander measured air sac volumes of 140 ml kg^−1^ in gentoo penguins (5–6 kg) with an unspecified technique [31]. Air sac volumes in Humboldt penguins (3–5 kg) have been estimated from three dimensional reconstructions based on serial CT scans [20]. For an average weight of 4.1 kg, air sac volume was 85 ml kg^−1^ during spontaneous ventilation of awake, minimally restrained birds. Differences between the two measurements in similarly sized species may be secondary to species, technique, body position, restraint and depth of inspiration. The CT technique appeared accurate as the lung volume in the study was similar to that previously determined by injection cast in the same species. Depth of inspiration was probably most important and is especially critical since penguins dive on inspiration, usually with the largest breaths prior to a dive [32].

In order to estimate the air sac volume on maximal inspiration prior to a dive, air sac volumes of Adélie, king and emperor penguins were determined during anaesthesia at 30 cm H_2_O fixed inflation pressure [21] (figure 2). This pressure was selected from a range of inflation pressures because the birds did not appear overdistended at that pressure, and body density/buoyancy calculations were reasonable. The volumes listed in table 1 resulted in mass specific air sac volumes of 296, 357 and 363 ml kg^−1^ in Adélie, king and emperor penguins, respectively (table 2). In comparison, the air sac volume of the awake, spontaneously breathing Humboldt penguin was about one-fifth that of the similarly sized Adélie penguin inflated at 30 cm H_2_O pressure. These differences again demonstrate how the compliance of the air sacs complicate the determination of how large a breath a penguin inspires prior to a dive.

**Table 2.** Estimated air volumes in three penguin species based on three different techniques. In simulated dives in a pressure chamber, the diving air volume (DAV) was determined during initial compression. The DAV includes respiratory air, any gastrointestinal gas and sub-feather air; the latter was determined to be 15 ml kg^−1^ (about 10% DAV) in Adélie penguins [16]. Buoyancy–velocity calculations at the ends of dives allowed calculation of the DAV in free-swimming birds; the DAV varied with maximum dive depth, maximum values are listed in the table. The lung–air sac–tracheal volumes were determined by computerized tomographic scan reconstructions from anaesthetized birds; air volume in the lung was calculated with the only relative lung morphometry data available—from the Humboldt penguin [22].

	species			reference
air volumes (in ml kg^−1^)	Adélie	king	emperor	
DAV simulated dive	165	69	—	[16,17]
DAV buoyancy–velocity	200	125	117	[33,34]
lung–air sac–tracheal air at 30 cm H_2_O inflation pressure	315	372	378	[21]
air sac volume at 30 cm H_2_O inflation pressure	296	357	363	[21]

#### (iii) ‘Diving’ air volumes

For comparison with these CT scan studies and evaluation of how much air a penguin inhales prior to a dive, we review estimations of air volumes from earlier studies during simulated dives in a pressure chamber and from studies during dives at sea. In the earliest studies, estimates of air volumes of 165 ml kg^−1^ in gentoo and Adélie penguins and 69 ml kg^−1^ in king penguins were made during simulated dives in a pressure chamber [16,17]. These ‘diving air volumes’ (DAVs), based on measurements during initial compression in the chamber, included respiratory air, any gas in the gastrointestinal system and sub-feather air, the latter of which was determined to be about 15 ml kg^−1^ in Adélie penguins. These DAVs were about 20% greater than the allometrically predicted total respiratory volume in gentoo and Adélie penguins, but 46% less than that predicted for king penguins. In addition, the gentoo and Adélie values were similar to that determined in gentoos by Scholander in 1940 [31].

Diving air volumes of Adélie and king penguins in dives at sea, determined by buoyancy–velocity calculations during final ascent [33], varied with maximum depth of dive and were greater than those determined in the pressure chamber (table 2). This raised the possibility that restraint during the simulated dives may have limited the inspiration prior to the submersion; consequently, the DAVs determined at sea have become those most widely used in estimating the size of the respiratory O_2_ store in penguins. A subsequent at-sea study of emperor penguins also demonstrated that DAV increased with maximum dive depth until about 300 m and then declined for deeper dives [34]. The DAVs determined at sea in these three species are all considerably less than the air sac and total respiratory air volumes determined by the CT scan studies (table 2). Two possible explanations are related to the time in the dive (final ascent) at which the DAVs were determined. It is possible that either exhalation of air underwater during the dive or absorption of respiratory gases into the body during the dive occurs [35]. Determinations of DAV during early descent would be helpful.

At this time, DAVs determined by buoyancy–velocity calculations in dives at sea are the most common values used in estimation of penguin respiratory O_2_ stores in the literature. However, as will be seen in the following discussion, these values are only 63%, 34% and 31% of the total respiratory air volumes determined by CT scan in Adélie, king and emperor penguins, respectively (table 2), and are too small to account for baroprotection during the deepest dives each species performs [21]. The size of the DAV prior to dives, especially deep dives in emperor penguins, remains unresolved. Further investigation is relevant not only to determine the mechanisms of baroprotection but also the magnitude of the respiratory O_2_ store. If the air sac volumes from CT studies were used instead of the DAV at sea, the total body O_2_ stores of the emperor penguin would increase from 68 to 119 ml kg^−1^, and the respiratory fraction of body O_2_ stores would increase from 33% to 61% of the total store [21].

### (b) Possible mechanisms of baroprotection

#### (i) Barotrauma defined

Before considering the protection of the penguin lung against increased hydrostatic pressure at depth, it is necessary to consider the concept and mechanism of pulmonary barotrauma. We note that the pathophysiology of barotrauma in humans is still incompletely understood, and may possibly be associated with alveolar collapse, capillary stress failure and/or alveolar re-expansion on ascent [36]. Pulmonary barotrauma has classically been considered to develop in human breath-hold divers when intrapulmonary pressure becomes less than the ambient hydrostatic pressure [37–39]. During descent, the air in the respiratory system decreases in volume with the increase in ambient pressure according to Boyle’s law (pressure × volume = constant). In addition, the decrease in air volume is partially compensated by a shift of blood into the chest and lungs from peripheral tissues compressed by the increased hydrostatic pressure. The decrease in lung air volume continues as far as the stiffness (compliance) of the thorax allows. Eventually, as descent continues to greater depths, the intrathoracic pressure is less than ambient pressure, and this results in ‘lung squeeze’ and atelectasis (alveolar collapse). The difference in transpulmonary pressure can result in pulmonary oedema (fluid accumulation in lung tissue), fluid extravasation (membrane damage and leakage of fluid into air spaces), and in haemorrhage from distention and rupture of engorged blood capillaries. In humans, lesions can occur in the alveoli of the lung and in the tracheobronchial tree [38,40–42].

#### (ii) Potential for barotrauma in penguins

In penguins, the respiratory air is primarily located in the air sacs and lungs. For now, we will omit consideration of the air in the tracheobronchial tree in order to simplify the discussion. The air space (air cavity) in the lungs has classically been considered fixed (i.e. rigid, immobile) while the air sacs have been considered much more compliant (although possibly limited by the compliance of the body wall). Consequently, compression of the air sac air into the ‘rigid’ lung space is at least initially protective against barotrauma. If the lung is rigid, it has a constant volume, meaning the air compressed from the air sacs into the lung will increase the lung pressure (Boyle’s law), leading to pressure equilibration. However, once all the lung + air sac air at depth has been compressed into the fixed air space of the lung, pressure within the fixed lung space will become less than ambient pressure as the penguin descends further. This decreased pressure in the ‘rigid’ air segments of the lung could result in barotrauma. The decreased pressure of the lung air cavity relative to pressure in the interstitial tissues and blood predisposes to pulmonary oedema, membrane rupture, fluid extravasation and capillary over-distention with rupture/haemorrhage. Dependent on the anatomy and compliance of the tracheobronchial tree (presumably less than that of the lungs but greater than that of the air sacs), it too, may be subject to barotrauma.

#### (iii) Mechanisms of baroprotection in penguins

With this as background, there are several potential mechanisms of protection against barotrauma in penguins at maximum depths. First is the ratio of collapsible air volume to ‘fixed’ space in the respiratory system. This protects against barotrauma to the extent that there is enough air in the compressible air spaces that can be transferred into the fixed air spaces, increasing fixed air space pressure until it equals the hydrostatic pressure at the deepest depth. This is primarily dependent on the volume of the highly compressible air sacs relative to the fixed volume of air space within the lungs. The larger the air sac volume and the smaller the fixed space volume, the deeper the depth threshold for barotrauma. This ratio is also affected by the compressibility (compliance) of the air sacs, tracheobronchial tree and the air components of the lung (parabronchi and air capillaries).

The most critical air volume regards barotrauma in the penguin lung is that of the air capillaries, the site of gas exchange [43,44]. Collapse of the air capillaries, although they have blind endings (closed at one end) [45], has long been considered unlikely because of the high probability that they would not re-expand due to their small diameter and radius of curvature [2,46,47]. In addition, a decrease in air capillary volume secondary to thoracic pooling of blood is not likely given recent investigations of the compliance and architecture of avian blood capillaries [48–50]. By contrast to the ‘fixed’ volume of the air capillaries, the parabronchi and tracheobronchial tree may be more compliant. Thoracic blood pooling into venous sinuses in the walls of these airways may also decrease their volume and contribute to baroprotection. Large venous sinuses have been documented in the tracheobronchial tree of other diving vertebrates [51–53], but histological examinations have not been performed in the trachea, bronchi or parabronchi of penguins. Tracheal volumes of other vertebrates can also decrease at depth dependent on the compliance of the tracheal wall [10,54], but biomechanical studies of penguin airways are needed for such evaluation. Another mechanism of baroprotection in penguins may be facilitated by constriction of the parabronchi. Smooth muscle cells and autonomic fibres underlie the parabronchial endothelium [2,25,55], and parasympathetic stimulation via the vagus nerve does constrict the parabronchi of ducks and geese [56,57]. Such vagal stimulation is feasible given the vagally mediated severe bradycardias in deep-diving emperor penguins [58].

In summary, the non-compliant air capillaries are the most critical and susceptible volume for barotrauma, while the air volumes of the parabronchi and tracheobronchial tree may be reduced by compression (biomechanics), blood engorgement (thoracic pooling) and vagally induced smooth muscle constriction in the parabronchi. Consequently, it is the ratio of air sac volume to air capillary volume rather than air sac volume to lung air volume that is probably most critical to depth thresholds for barotrauma in penguins.

#### (iv) Modelling baroprotection

The depth threshold at which barotrauma occurs in diving penguins can be modelled by calculating the ratio of the compressible air space to the fixed air space. As discussed above, the compliance and volumes of compressible versus fixed air space are not yet clearly defined. If one assumes that air sac air compresses into lungs and airways that are completely rigid and do not change with depth, the calculated depth limits for baroprotection in penguins are inadequate [21]. These depth limits were initially estimated to be 153, 221 and 230 m in Adélie, king and emperor penguins, respectively, based on compression of the maximal inspiratory air sac volume (30 cm H_2_O (2.94 kPa) inflation pressure) determined by the CT scan measurements to a volume less than that estimated morphometrically in the lungs and tracheobronchial tree [21]. A more precise estimation of baroprotection depth limits would utilize the total respiratory air volume (lung air space volume, extrapulmonary tracheobronchial volume and air sac volume; see table 1*a*) instead of the air sac volume alone [31]. However, the resulting barotrauma depth thresholds would only increase to 161, 231 and 240 m, respectively, due to the relatively large size of the air sac volumes. These depth limits are still too low for baroprotection during the deepest dives (180 m, 343 m and 564 m) reported for Adélie, king and emperor penguins, respectively [59–61]. Some reduction in the ‘fixed’ air space of the lungs and airway seems necessary.

As discussed above, collapse of the air capillaries is unlikely. Consequently, we have calculated the percentage reduction of the combined parabronchial–tracheobronchial tree space that would be required to provide baroprotection for the deepest dives of Adélie, king and emperor penguins (see table 1*b*). Reductions of the combined parabronchial–tracheobronchial tree space of 24% in Adélie penguins, 53% in king penguins and 76% in emperor penguins would be required to provide baroprotection limits of 200, 400 and 600 m, respectively.

These calculations represent our current best explanation of how barotrauma is avoided in deep-diving penguins. However, these conclusions are dependent on many assumptions that require exploration. Perhaps most importantly, the respiratory air volumes determined by the CT scan study used in this calculation are much greater than DAVs determined by buoyancy–velocity calculations at sea. These discrepancies need resolution. In addition, lung morphometry of deep-diving penguins may be significantly different from that in the shallow-diving Humboldt penguin. Smaller parabronchial and air capillary volumes would promote baroprotection in deeper divers. Accurate determination of the start-of-dive respiratory air volume, lung morphometry, and biomechanical and histological investigation of the tracheobronchial tree, parabronchi and air capillaries are needed. One other limitation of the buoyancy–velocity calculation of air volume at the end of a dive is that absorption of gases from the respiratory system into the blood at depth is not considered. Although this effect may be small, both O_2_ and nitrogen (N_2_) diffuse into the blood at depth, and the diffusion of carbon dioxide from blood into the lung can be blunted and even reversed because of the high partial pressure of carbon dioxide at depth [16,35,62]. Consequently, an end-of-dive calculation may underestimate the start-of-dive air volume.

## 3. Risks of decompression sickness and nitrogen narcosis

Mechanisms of avoidance of excess nitrogen (N_2_) absorption and minimization of the risks of decompression sickness and nitrogen narcosis are unresolved in penguins. Alveolar collapse in marine mammals minimizes gas exchange at depth [11,63], which results in less N_2_ absorption into the blood or tissue reducing the risk of decompression sickness and nitrogen narcosis. However, there is no evidence that gas exchange ceases in penguins at depth. If most of the respiratory air volume is compressed into the air capillaries, gas exchange and N_2_ absorption will continue throughout the deepest dives. In simulated dive studies in a pressure chamber, blood N_2_ levels continued to increase during the deepest dives in the studies, 68 m in Adélie and gentoo penguins and 136 m in king penguins [16,17].

Here, we briefly consider how excess N_2_ absorption may be avoided or at least minimized in the deepest of avian divers, the emperor penguin. Deep dives (>400 m) are typically 8–10 min long, with bottom phases of only 2–3 min [8,9,34,61]. A short time at depth minimizes the time for N_2_ uptake. Deep dives often have long inter-deep-dive surface intervals (IDDI: time between deep dives, it includes shallow dives and surface periods). In one study, 41% of deep dives had IDDIs > 30 min, and in the remaining 90 deep dives, IDDIs ranged from 4 to 29 min with a mean of 15.8 min [9]. Such long IDDIs maximize time both for metabolic recovery and N_2_ washout after these deep dives [15,64].

Nitrogen uptake from the lungs into the blood during the dive would also be minimized by the low heart rates and low cardiac output at greatest depths (i.e. 17 beats min^−1^ during bottom phase of a 423 m dive [58]) and by the short duration of bottom phases of deep dives [9,58,65]. A decreased volume of distribution for N_2_ associated with the bradycardia, low cardiac output and widespread vasoconstriction of the dive response in deep dives would also result in less N_2_ loading into the body. Such restriction of peripheral tissue flow would result in decreased flux of blood N_2_ into tissue depots and result in return of blood with higher N_2_ content to the lung, decreasing the gradient for N_2_ uptake into the blood. Possible arterio-venous shunting during dives could do the same [66]. Conversely, the increasing heart rate during ascent and the associated increase in lung and tissue perfusion could promote the lowering of blood N_2_ levels during ascent through the distribution of N_2_ to previously vasoconstricted tissues as well as through the transfer of N_2_ back into the lungs as the respiratory partial pressure of N_2_ decreases with the decline in ambient pressure [16,17]. Although the dive response and a decreased volume of distribution may result in a net decrease in tissue N_2_ loading at depth, it has the potential to elevate arterial N_2_ levels at depth, resulting in elevated brain N_2_ levels and increased risk of N_2_ narcosis. Our understanding of how the risks of decompression sickness and nitrogen narcosis are avoided in deep-diving penguins requires further investigation.

## 4. Air movement between air sacs and lungs during breathing and during diving

Unidirectional air flow through the lungs during the avian respiratory cycle is accomplished through complex air movements in the lungs and air sacs during inhalation and exhalation [45,55,67]. These respiratory air flow patterns provide movement of air through the parabronchi into the air capillaries in the lung, the sites of gas exchange with the blood. As in other birds, the air sacs of penguins are classified as anterior (cervical, interclavicular and anterior thoracic air sacs) and posterior (posterior thoracic and abdominal air sacs). During inspiration, ambient air enters the trachea and flows into the posterior air sacs and lungs while air from the lungs moves into the anterior air sacs. During expiration, anterior air sac air exits the body via the trachea, while lung air flows into the anterior air sacs and posterior air sac air enters the lungs. Consequently, the partial pressure of O_2_ (P_O2_) in the posterior air sacs is typically near that of inspired air while the P_O2_ in the anterior air sacs is usually 20−30 mm Hg (2.7−4.0 kPa) less due to O_2_ extraction in the lungs [67–69]. P_O2_ profiles in the cervical and posterior thoracic air sacs of emperor penguins on sea ice are consistent with such air flow patterns during the breathing cycle [70,71]. At rest, cervical air sac P_O2_s are about 50 mm Hg (6.7 kPa) less than that in the posterior thoracic air sacs, presumably secondary to slow ventilatory rates and greater blood O_2_ extraction in the lungs of emperor penguins [71].

**Figure 3.**
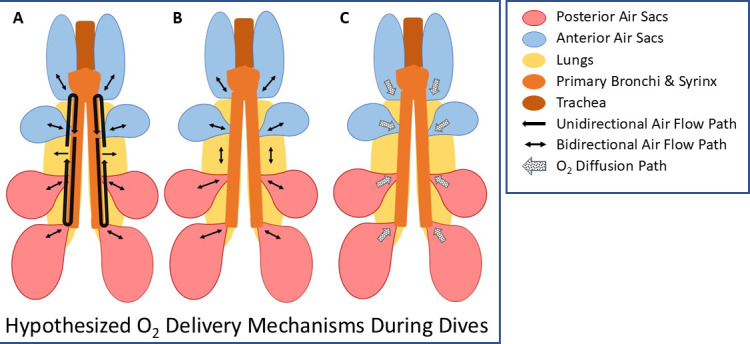
Hypothesized air movement patterns within the respiratory system during dives of penguins. As hydrostatic pressure increases with depth during a dive, compression of air sacs results in transfer of air into the parabronchi and air capillaries of the lung. During ascent, decreased hydrostatic pressure results in expansion of the air sacs. In addition, movement of air through the parabronchi may occur secondary to differential air sac pressures induced by wing movements [74]. Unidirectional movement of air through the parabronchi could result from air routing from anterior air sacs through the primary bronchi into posterior air sacs and then into the lung with return to the anterior air sacs (*a*). Bidirectional movement of air through the parabronchi could result from to and fro movement between the anterior and posterior air sacs via secondary bronchi and lungs (*b*). Lastly, even without significant convective air transport, enhanced diffusion of oxygen into the parabronchi and air capillaries from the air sacs (via secondary bronchi) could result from differential air sac pressure oscillations as well as the increase in the partial pressure of oxygen within the air sacs and lungs (*c*). Modified from Williams *et al.* [71].

During diving, however, breathing stops. Yet, gas exchange continues as evidenced by arterial and air sac oxygen profiles in diving emperor penguins and by increased blood N_2_ levels in Adélie, gentoo and king penguins during simulated dives as deep as 136 m in pressure chambers [16,17,70–73]. Compression of air sac air into the lungs and elevation of P_O2_ at depth have long been considered to contribute to gas exchange during dives [16,17,21,70–73]. But are these two mechanisms sufficient for adequate gas exchange in a dive especially at shallower depths when most of the respiratory air volume is still in the air sacs? Or are other mechanisms of mixing or moving air through in the parabronchi needed to enhance gas exchange (figure 3)?

In the 1990s, Boggs and co-workers investigated the effect of air sac pressure changes associated with locomotion in supplementing movement of air between air sacs and lungs during the respiratory cycle [75]. In a study of little blue penguins swimming underwater in a flume, small differential pressure oscillations (0.06 kPa) between the anterior and posterior air sacs were associated with their rapid (3 Hz) wing movements [74]. It was postulated that these oscillations enhanced air flow (possibly bi-directional [76]) through the parabronchi, thus promoting access to and utilization of the air sac O_2_ stores during the breath hold. Although the postulated convective air movements were small and interactions with aerodynamic valving in the airways were unknown, Boggs *et al*. hypothesized that these small, rapid oscillations optimized O_2_ diffusion, analogous to high frequency positive pressure ventilation techniques (mechanical ventilation with small tidal volumes and rapid ventilatory rates) [77,78]. These authors suggested that this mechanism may be especially important in penguins with high wing beat frequencies during sub-surface swimming and shallow dives because depth-related air sac compression into the lungs and elevation of air sac P_O2_ are relatively less in these situations than in deep dives. In this regard, penguins species with smaller body masses are typically shallow divers and have higher dominant stroke frequencies in the 2−3 Hz range [79].

**Figure 4.**
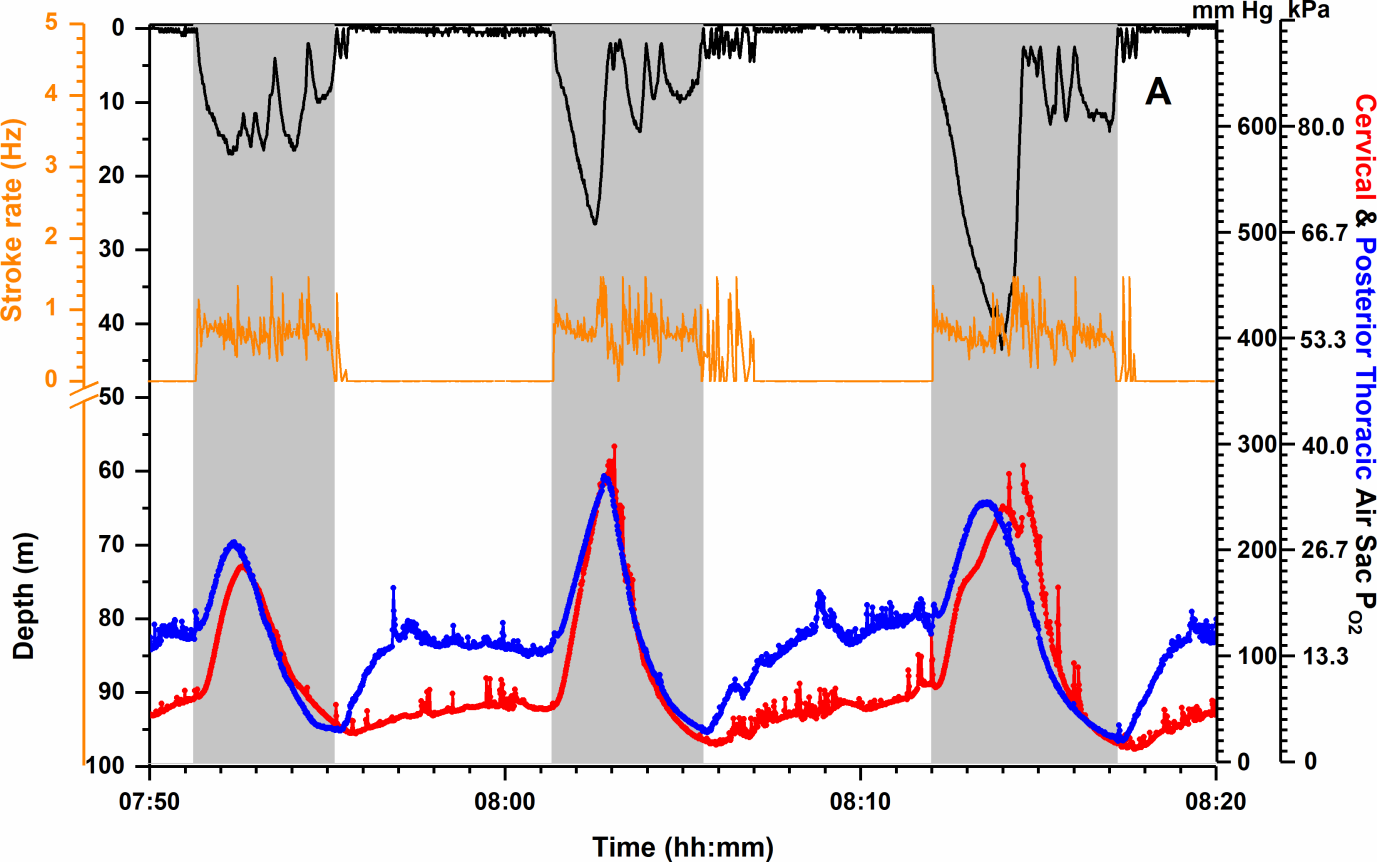
Cervical and posterior thoracic air sac partial pressure of O_2_ (P_O2_) profiles with depth and stroke rate profiles during three shallow dives of an emperor penguin [71]. The merging and overlap of air sac P_O2_ profiles could occur at different points in a dive but did not correlate with depth, time into dive or stroke rate. In addition, overlap did not occur in all dives. The shapes of air sac P_O2_ profiles can be dependent on many factors, including start-of-dive respiratory air volumes, O_2_ consumption during diving, changes in air sac P_O2_ secondary to changes in depth, the rate of O_2_ diffusion, the compression of air sac air into the lung on descent and re-expansion of that air into the air sacs on ascent, and possible differential air sac pressure oscillations due to wing movement [71,74,80]. See text and electronic supplementary materials. Modified from Williams *et al.* [71].

In order to further evaluate the potential for differential air sac pressure oscillations to promote air movement in the lungs in diving penguins (see figure 3), Williams *et al*. examined cervical and posterior thoracic air sac P_O2_ profiles in diving emperor penguins [71]. Although highly variable, these profiles demonstrated that (i) P_O2_ in both air sacs increased during descent and decreased during ascent, (ii) the difference between cervical and posterior thoracic P_O2_s usually declined during descent, and (iii) the two profiles could even merge as early as late descent and then overlap during much of the dive (figure 4). Such overlap was taken as evidence of mixing of air within the lung during dives, but the merging and overlap of the two profiles had no relation to number of wing beats, depth or time into dive. The data provided no evidence for or against the hypothesis that wing beat induced oscillations in air sac pressures resulted in parabronchial air movement within the lung. In addition, wing beat frequency of emperor penguins was much less than in little blue penguins, typically less than 1.5 Hz with mean values closer to 0.5 Hz [71,81]. Furthermore, any decrease with depth in the diameter of parabronchi and primary bronchi within the lung may increase resistance to flow induced by air sac pressure oscillations. Although the mechanism remained unclear, it was concluded that the air sac O_2_ profiles were consistent with air movement/mixing within parabronchi and air capillaries of the lungs.

More recently, on review of the data of Williams *et al*. [71], Ainley and Wilson have emphasized that the change in depth during a dive is the primary regulator of air sac O_2_ utilization [80]. Essentially, these authors postulated that air movement within the parabronchi and air capillaries (as proposed by Boggs *et al*. [74]) was not essential for gas exchange during the dive. The addition of the compressed air volume from the air sacs and the elevated P_O2_ were considered sufficient to enhance O_2_ diffusion into the blood. They concluded that ‘greater depths should allow penguins to access more of their air sac oxygen stores’ and that ‘deep-diving penguins would remove proportionately more oxygen from their air sacs’. However, it is unclear if these processes alone account for adequate gas exchange.

We base our concerns about the limitations of this hypothesis on the simulated dive study of gas exchange in Adélie and gentoo penguins by Kooyman and co-workers [16]. In that study, thoracic air sac O_2_ contents were determined by analysis of syringe-drawn air samples during simulated dives in a pressure chamber both at sea level (1 atmosphere absolute, ATA) with no compression and at 30 m depth (4 ATA). In the control 2.5 min simulated dive (sea level), air sac O_2_ content decreased 66% or 26% per minute from 13.2% to 4.5%. This large decline occurred despite no depth-related compression of air sac air into the lung or elevation in air sac P_O2_. Most of the air in the respiratory system (92%) was in the air sacs. Clearly, in this situation, processes other than compression of air into the lungs and elevation of air sac P_O2_ contributed to gas exchange. Notably, during these studies, the penguins had rapid wing beats throughout the simulated dives (GL Kooyman, 2023, personal communication). Interestingly, during a simulated dive to 30 m depth, the air sac O_2_ fraction declined 85% over 3.5 min (about 25% min^−1^, similar to the simulated dive at sea level). We conclude that differential air sac pressure oscillations associated with wing beats may yet have a role in supplementing gas exchange during dives.

In addition, we suspect that the net transfer of air sac air into the lung with depth is probably slower than proposed by Ainley and Wilson because the lung volume used in their calculations was not the air volume within the lung, but rather the total tissue + air volume of the lung [21,80]. Based on the only available lung morphometry data in penguins, air volume (primary bronchi within the lung, parabronchi, air capillaries) in the lung is only about 59% of total lung air + tissue volume [19,22]. The quantity of air transferred from the air sacs into the lungs for a given change in depth would be less than in their calculations (see electronic supplementary material). The depth at which all air sac air would be transferred into the lung would also be deeper than in their calculations. Consequently, further analyses and investigation of gas exchange at depth are needed to resolve these questions.

In reviewing these papers, we also realized that the process of a gradual inflow of air with an elevated P_O2_ from an air sac reservoir into the parabronchi of the lung during descent is somewhat analogous to apnoeic oxygenation techniques for human patients during surgery [82]. During apnoeic oxygenation (essentially a prolonged breath hold on 100% O_2_), blood oxygenation is maintained by diffusion of O_2_ into blood in the alveolus and the bulk movement of O_2_ down the tracheobronchial tree into the alveolus from an O_2_ reservoir in the anaesthesia breathing circuit. There is no convective O_2_ delivery. This technique provides adequate O_2_ delivery for a low metabolic rate during anaesthesia. However, a buildup of carbon dioxide does occur in the blood. A similar process occurs in many hibernating animals [83]. Apnoeic O_2_ diffusion down an open glottis, in combination with a reduced metabolism and shift in the O_2_–haemoglobin dissociation curve allows apnoeas as long as 45 min. Carbon dioxide accumulates during these apnoeas and is eliminated by intermittent bouts of breathing. Although speculative, it is conceivable that, with depth-related elevations in air sac P_O2_, this type of apnoeic diffusion of O_2_ from the air sac to the air capillary could also contribute to gas exchange in a diving penguin even without compression or movement of air sac air into the lungs. Such a mechanism could blunt the depth-related rise in air sac P_O2_ during descent, but whether that mechanism alone is sufficient to maintain oxygenation in an actively swimming penguin (especially at shallow depths) is unclear.

Lastly, we point out that interpretation of air flow patterns within the lung from air sac P_O2_ profiles during dives is complex (see electronic supplementary materials). As pointed out in the original paper on air sac P_O2_ in diving emperor penguins [70], these limitations are primarily based on the response time of the P_O2_ electrode (1 min, 90% response time). This slow response time is especially relevant for air sac P_O2_ because air sac P_O2_ changes instantaneously with depth, and the rates of vertical changes in depth of penguins are often rapid. In addition, the electrode is probably less accurate and subject to more drift at extremely high P_O2_ values [70]. Interpretation is also limited by the assumption of uniform mixing of oxygen within the air sacs during a dive, the unknown location of the P_O2_ electrode within a given air sac, and probable differences in start-of-dive air sac volumes for dives to different depths. In conclusion, the mechanism(s) of gas exchange at depth require more investigation.

## 5. Air sac and arterial partial pressure of oxygen at rest, and prior to and during dives— implications for gas exchange

In this section, we review possible explanations as to why arterial P_O2_ of emperor penguins at rest is lower than that in most birds, and why peak arterial P_O2_ during dives is typically less than that in the air sac. Despite lower than expected arterial P_O2_, arterial haemoglobin saturation is above 90% both at rest, and during most of the dive in part because of the higher affinity of haemoglobin in emperor penguins and, during dives, because of the elevated air sac and arterial P_O2_ at depth [66,84].

### (a) Partial pressure of oxygen at rest

The arterial P_O2_ of 68 mm Hg (9.1 kPa) in emperor penguins at rest [73], determined both by blood sample analysis and by indwelling P_O2_ electrodes, was in the lower range of most birds, and certainly less than values near 95 mm Hg (12.7 kPa) in most waterfowl, bar-headed geese and smaller penguin species [85–89]. Anterior air sac P_O2_ in emperor penguins at rest was also lower than in most birds at rest (50 mm Hg (6.7 kPa) versus 80–100 mm Hg (10.7–13.3 kPa)) [67–69,71]. Posterior air sac values at rest were typically 110–130 mm Hg (14.7 – 17.3 kPa) in emperor penguins [70]. Notably, the difference between simultaneous anterior and posterior air sac P_O2_s in an emperor penguin was 60 mm Hg (8.0 kPa), about two to three times the difference in other birds [67,69,71].

Williams *et al*. suggested that these findings were consistent with the low respiratory rates of emperor penguins at rest [90] resulting in a low ratio of parabronchial ventilation to parabronchial blood O_2_ extraction [71]. That ratio is also dependent on tidal volume, heart rate (cardiac output) and the P_O2_/O_2_ content of mixed venous blood returning to the lung. Ventilation was considered most likely because heart rate at rest is near allometric predictions and venous blood P_O2_ at rest is unremarkable [73,90]. A diffusion limitation or a pulmonary shunt could also cause a low arterial P_O2_ but in these cases, the P_O2_ difference between the anterior air sac and artery should approach 0 or even become positive [91,92]. The large negative P_O2_ difference between the mean arterial and cervical air sac was not consistent with these two processes. However, some limitation of diffusion is suggested by the increased thickness and small surface area of the blood–gas barrier in Humboldt penguins [22,25]. Lastly, a contribution to a low arterial P_O2_ from a ventilation–perfusion inhomogeneity could not be ruled out because it can result in a negative difference between the arterial and anterior air sac P_O2_ [91].

### (b) Partial pressure of oxygen prior to dives

In general, cervical air sac and arterial P_O2_, increase relative to resting levels prior to diving activity of emperor penguins [71–73] (see figure 2). These elevations in P_O2_ are probably secondary to an increased ratio of parabronchial ventilation to parabronchial blood O_2_ extraction due to the pre-dive tachycardia and hyperventilation observed in penguins prior to the start of dives [32,90]. This hyperventilation hypothesis was originally proposed to account for elevations in anterior air sac P_O2_ during the hyperventilation of exercising domestic fowl [68]. Pre-dive arterio-venous shunting and arterialization of venous blood may also contribute to a relative decrease in parabronchial blood O_2_ extraction and an elevation in anterior air sac P_O2_ [66,84]. Hyperventilation and an elevated cardiac output presumably also contribute to the recovery of air sac and arterial P_O2_ during the surface interval between dives.

### (c) Partial pressure of oxygen during dives

Air sac and arterial P_O2_ profiles during dives increase during descent and decrease during ascent. The net depletion of the respiratory and blood O_2_ stores, dependent on metabolic rate and the duration of a dive, have been reviewed elsewhere [66,71,93] and are not the focus of this review. Here we consider the difference between air sac and arterial P_O2_ during dives.

Although simultaneous air sac and arterial P_O2_ profiles have not been collected during dives of emperor penguins, peak arterial P_O2_ values are typically much less than peak air sac values during dives to shallow depths [66,70–73]. Start-of-dive arterial P_O2_s are usually closer to posterior air sac values, while end-of-dive arterial values, although considerably variable, can approximate values in the air sacs [70–73]. During simulated dives of Adélie and gentoo penguins in a pressure chamber [16], pre-dive and intra-dive arterial P_O2_s were also less than those in the thoracic air sac, although differences were less than those found in emperor penguins.

It is unclear why there is such a large difference between peak arterial and air sac P_O2_s during dives in emperor penguins. Arterial P_O2_ is dependent on air sac P_O2_ (ambient pressure and the respiratory O_2_ fraction), heart rate (cardiac output), the P_O2_ and haemoglobin saturation of mixed venous blood returning to the heart (lower with increased metabolic rate and higher with peripheral arterio-venous shunting) and the adequacy of O_2_ diffusion from the air capillary into the blood (O_2_ flux). A diffusion limitation secondary to a thickened blood–gas barrier with a decreased surface area [22,25] may limit O_2_ flux but, as discussed above, this was considered unlikely in penguins at rest on the basis of the difference between cervical air sac and arterial P_O2_. Interpretation of differences in air sac and arterial P_O2_ profiles in regard to ventilation–perfusion matching and pulmonary shunts is also difficult because of the unknown pattern of mixing or movement within the parabronchi and air capillaries at depth. Arterial haemoglobin saturations greater than 95% during most of even a 10 min dive in emperor penguins suggest a significant pulmonary shunt is unlikely during dives [66,84].

It remains to be determined whether elevated air sac P_O2_ and gradual compression of air sac air into the lung with depth provide sufficient mixing of air within the lung, or whether additional movement of air induced by differential air sac pressures associated with wing movement or by some other mechanism are necessary for adequate diffusion of O_2_ into blood [71,74,80]. It may be possible that limitation of parabronchial air movement or mixing during a dive decreases the efficiency of gas exchange and accounts for the difference between peak arterial and air sac P_O2_s in diving emperor penguins.

## 6. Summary and future directions of research

Although our knowledge of penguin lung anatomy and lung function has increased greatly over the past 30 years, much speculation remains as to mechanisms of baroprotection and gas exchange at depth. In this summary, we return to the observations and prescient conclusions of Maina and King in their morphometric study of the Humboldt penguin lung [22]. Key observations included a markedly thickened blood–gas barrier with a decreased mass specific surface area, an increase in pulmonary capillary blood volume, and a reduction in the volume of the air capillaries. These findings resulted in a low mass specific anatomical O_2_ diffusion capacity for the blood–gas barrier, but yielded a total lung anatomical O_2_ diffusion capacity within the range of other birds. They concluded that the penguin lung was not as well adapted for O_2_ uptake as in flying birds, and that O_2_ consumption during swimming and diving was low relative to that during flight. Since that time, measurements of air sac-to-arterial P_O2_ differences at rest and during dives, of swimming metabolic rates, of foraging field metabolic rates at sea, and of blood/muscle O_2_ depletion rates during dives have been consistent with those conclusions [24,65,84,93–95].

We suspect that the lung morphometry of deeper-diving penguins will be even more extreme than in the Humboldt penguins. A thickened blood–gas barrier in penguins has already been considered to contribute to membrane stability during compression at depth [25]. We postulate that (i) thicker blood–gas barriers with decreased mass specific surface areas and (ii) larger increases in pulmonary capillary blood volume further decrease air capillary volume and improve pressure tolerance in deeper-diving penguins. In emperor penguins, such morphometry would be consistent with their deep dive behaviour, with the air sac-to-arterial differences in P_O2_, and with their relatively low swimming metabolic rates, field metabolic rates and O_2_ store depletion rates. Maintenance of O_2_ flux across a thickened blood–gas barrier may also be facilitated by the high O_2_ affinity of emperor penguin haemoglobin [66].

To address these and other questions raised in this review, we suggest the following: (i) more morphology/morphometry studies of the lungs in different species; (ii) investigation of lung and tracheobronchial anatomy with advanced imaging techniques (computerized tomography, magnetic resonance imaging); (iii) biomechanical and histological investigation of the tracheo-bronchial tree, parabronchi and air capillaries; (iv) determination of start-of-dive air sac volumes; (v) measurement of simultaneous P_O2_ profiles (anterior air sac–posterior air sac, air sac–arterial, arterial-mixed venous) with P_O2_ sensors with faster response times; and (vi) application of backpack blood samplers for arterial, venous and mixed venous blood gas analyses (oxygen, carbon dioxide, nitrogen).

## Data Availability

Supplementary material is available at [96].

## References

[1] Ponganis PJ . 2015 Diving physiology of marine mammals and seabirds. Cambridge, UK: Cambridge University Press. (10.1017/CBO9781139045490)

[2] Duncker HR . 1972 Structure of avian lungs. Respir. Physiol. **14** , 44–63. (10.1016/0034-5687(72)90016-3) 5042157

[3] Scheid P , Slama H , Willmer H . 1974 Volume and ventilation of air sacs in ducks studied by inert gas wash-out. Respir. Physiol. **21** , 19–36. (10.1016/0034-5687(74)90004-8) 4846935

[4] Bethge P , Nicol S , Culik BM , Wilson RP . 1997 Diving behaviour and energetics in breeding little penguins (Eudyptula minor). J. Zool. Lond. **242** , 483–502. (10.1111/j.1469-7998.1997.tb03851.x)

[5] Luna-Jorquera G , Culik BM . 1999 Diving behaviour of Humboldt penguins Speniscus humboldti in northern Chile. Mar. Ornithol. **27** , 67–76.

[6] Williams TD , Briggs DR , Croxall JP , Naito Y , Kato A . 1992 Diving pattern and performance in relation to foraging ecology in the gentoo penguin, Pygoscelis papua. J. Zool. Lond. **227** , 211–230. (10.1111/j.1469-7998.1992.tb04818.x)

[7] Pütz K , Wilson RP , Charrassin JB , Raclot T , Lage J , Le Maho Y . 1998 Foraging strategy of king penguins (Aptnodytes patagonicus) during summer at the Crozet Islands. Ecology **79** , 1905–1921. (10.2307/176698)

[8] Kooyman GL , Kooyman TG . 1995 Diving behavior of emperor penguins nurturing chicks at Coulman Island, Antarctica. Condor **97** , 536–549. (10.2307/1369039)

[9] Kooyman GL , Goetz K , Williams CL , Ponganis PJ , Sato K , Eckert S . 2020 Crary Bank: a deep foraging habitat for emperor penguins in the western Ross Sea. Polar Biol. **43** , 801–811. (10.1007/s00300-020-02686-3)

[10] Kooyman GL , Hammond DD , Schroeder JP . 1970 Bronchograms and tracheograms of seals under pressure. Science **169** , 82–84. (10.1126/science.169.3940.82) 5447537

[11] Kooyman GL , Schroeder JP , Denison DM , Hammond DD , Wright JJ , Bergman WD . 1973 Blood N_2_ tensions of seals during simulated deep dives. Am. J. Physiol. **223** , 1016–1020. (10.1152/ajplegacy.1972.223.5.1016) 4654334

[12] Fahlman A , Hooker SK , Olszowka A , Bostrom BL , Jones DR . 2009 Estimating the effect of lung collapse and pulmonary shunt on gas exchange during breath-hold diving: the Scholander and Kooyman legacy. Respir. Physiol. Neurobiol. **165** , 28–39. (10.1016/j.resp.2008.09.013) 18973832

[13] Falke KJ , Hill RD , Qvist J , Schneider RC , Guppy M , Liggins GC , Hochachka PW , Elliott RE , Zapol WM . 1985 Seal lungs collapse during free diving: evidence from arterial nitrogen tensions. Science **229** , 556–558. (10.1126/science.4023700) 4023700

[14] Kooyman GL , Sinnett EE . 1982 Pulmonary shunts in harbor seals and sea lions during simulated dives to depth. Physiol. Zool. **55** , 105–111. (10.1086/physzool.55.1.30158447)

[15] Fahlman A , Schmidt A , Jones DR , Bostrom BL , Handrich Y . 2007 To what extent might N_2_ limit dive performance in king penguins? J. Exp. Biol. **210** , 3344–3355. (10.1242/jeb.008730) 17872988

[16] Kooyman G , Schroeder J , Greene D , Smith V . 1973 Gas exchange in penguins during simulated dives to 30 and 68 m. Am. J. Physiol. Leg. Content **225** , 1467–1471. (10.1152/ajplegacy.1973.225.6.1467) 4760462

[17] Ponganis PJ , Kooyman GL , Van Dam R , LeMaho Y . 1999 Physiological responses of king penguins during simulated diving to 136 m depth. J. Exp. Biol. **202** , 2819–2822. (10.1242/jeb.202.20.2819) 10504317

[18] Lasiewski RC , Calder WA . 1971 A preliminary allometric analysis of respiratory variables in resting birds. Respir. Physiol. **11** , 152–166. (10.1016/0034-5687(71)90020-x) 5540203

[19] Maina JN , Nathaniel C . 2001 A qualitative and quantitative study of the lung of an ostrich, Struthio camelus. J. Exp. Biol. **204** , 2313–2330. (10.1242/jeb.204.13.2313) 11507114

[20] Nevitt BN , Langan JN , Adkesson MJ , Mitchell MA , Henzler M , Drees R . 2014 Comparison of air sac volume, lung volume, and lung densities determined by use of computed tomography in conscious and anesthetized Humboldt penguins (Spheniscus humboldti) positioned in ventral, dorsal, and right lateral recumbency. Am. J. Vet. Res. **75** , 739–745. (10.2460/ajvr.75.8.739) 25061705

[21] Ponganis PJ , St Leger J , Scadeng M . 2015 Penguin lungs and air sacs: implications for baroprotection, oxygen stores and buoyancy. J. Exp. Biol. **218** , 720–730. (10.1242/jeb.113647) 25740902

[22] Maina JA , King AS . 1987 A morphometric study of the lung of the Humboldt penguin (Spheniscus humboldti). Zentralblat Vet. Med. C **16** , C293–C297. 3434830

[23] Bennett PM , Harvey PH . 1987 Active and resting metabolism in birds: allometry, phylogeny and ecology. J. Zool. **213** , 327–344. (10.1111/j.1469-7998.1987.tb03708.x)

[24] Nagy KA , Kooyman GL , Ponganis PJ . 2001 Energetic cost of foraging in free‐diving emperor penguins. Physiol. Biochem. Zool. **74** , 541–547. (10.1086/322165) 11436138

[25] Welsch U , Aschauer B . 1986 Ultrastructural observations on the lung of the emperor penguin (Aptenodytes forsteri). Cell Tissue Res. **243** , 137–144.

[26] Hawkins MG , Malka S , Pascoe PJ , Solano AM , Kass PH , Ohmura H , Jones JH . 2013 Evaluation of the effects of dorsal versus lateral recumbency on the cardiopulmonary system during anesthesia with isoflurane in red-tailed hawks (Buteo jamaicensis). Am. J. Vet. Res. **74** , 136–143. (10.2460/ajvr.74.1.136) 23270358

[27] Malka S , Hawkins MG , Jones JH , Pascoe PJ , Kass PH , Wisner ER . 2009 Effect of body position on respiratory system volumes in anesthetized red-tailed hawks (Buteo jamaicensis) as measured via computed tomography. Am. J. Vet. Res. **70** , 1155–1160. (10.2460/ajvr.70.9.1155) 19719433

[28] King AS , Payne DC . 1964 Normal breathing and the effects of posture in Gallus domesticus. J. Physiol. **174** , 340–347. (10.1113/jphysiol.1964.sp007491) 14232397 PMC1368934

[29] Scheid P , Piiper J . 1969 Volume, ventilation and compliance of the respiratory system in the domestic fowl. Respir. Physiol. **6** , 298–308. (10.1016/0034-5687(69)90029-2) 5778476

[30] Stephenson R . 1995 Respiratory and plumage gas volumes in unrestrained diving ducks (Aythya affinis). Respir. Physiol. **100** , 129–137. (10.1016/0034-5687(94)00130-r) 7624614

[31] Scholander PF . 1940 Experimental investigations on the respiratory function in diving mammals and birds. Hvalradets Skr. **22** , 1–131.

[32] Wilson RP , Simeone A , Luna-Jorquera G , Steinfurth A , Jackson S , Fahlman A . 2003 Patterns of respiration in diving penguins: is the last gasp an inspired tactic? J. Exp. Biol. **206** , 1751–1763. (10.1242/jeb.00341) 12682106

[33] Sato K , Naito Y , Kato A , Niizuma Y , Watanuki Y , Charrassin JB , Bost CA , Handrich Y , Le Maho Y . 2002 Buoyancy and maximal diving depth in penguins. J. Exp. Biol. **205** , 1189–1197. (10.1242/jeb.205.9.1189) 11948196

[34] Sato K , Shiomi K , Marshall G , Kooyman GL , Ponganis PJ . 2011 Stroke rates and diving air volumes of emperor penguins: implications for dive performance. J. Exp. Biol. **214** , 2854–2863. (10.1242/jeb.055723) 21832128

[35] Fahlman A , Sato K , Miller P . 2020 Improving estimates of diving lung volume in air-breathing marine vertebrates. J. Exp. Biol. **223** , b216846. (10.1242/jeb.216846) 32587107

[36] Patrician A , Spajić B , Gasho C , Caldwell HG , Dawkins T , Stembridge M . 2021 Temporal changes in pulmonary gas exchange efficiency when breath-hold diving below residual volume. Exp. Physiol. **106** , 1120–1133. (10.1113/EP089176) 33559974

[37] Mijacika T , Dujic Z . 2016 Sports-related lung injury during breath-hold diving. Eur. Respir. Rev. **25** , 506–512. (10.1183/16000617.0052-2016) 27903671 PMC9487548

[38] Lindholm P , Lundgren CE . 2009 The physiology and pathophysiology of human breath-hold diving. J. Appl. Physiol. **106** , 284–292. (10.1152/japplphysiol.90991.2008) 18974367

[39] Adir Y , Bove AA . 2014 Lung injury related to extreme environments. Eur. Respir. Rev. **23** , 416–426. (10.1183/09059180.00006214) 25445940 PMC9487397

[40] Lindholm P , Ekborn A , Öberg D , Gennser M . 2008 Pulmonary edema and hemoptysis after breath-hold diving at residual volume. J. Appl. Physiol. **104** , 912–917. (10.1152/japplphysiol.01127.2007) 18202166

[41] Lindholm P , Norris CM , Braver JM , Jacobson F , Ferrigno M . 2009 A fluoroscopic and laryngoscopic study of glossopharyngeal insufflation and exsufflation. Respir. Physiol. Neurobiol. **167** , 189–194. (10.1016/j.resp.2009.04.013) 19383557

[42] Tetzlaff K , Lemaitre F , Burgstahler C , Luetkens JA , Eichhorn L . 2021 Going to extremes of lung physiology—deep breath-hold diving. Front. Physiol. **12** , 710429. (10.3389/fphys.2021.710429) 34305657 PMC8299524

[43] Maina JM . 2006 Development, structutre, and function of a novel respiratory organ, the lung-air sac system of birds: to go where no other vertebrate has gone. Biol. Rev. **81** , 5455–5579. (10.1017/S1464793106007111) 17038201

[44] Maina JN *et al* . 2010 Recent advances into understanding some aspects of the structure and function of mammalian and avian lungs. Physiol. Biochem. Zool. **83** , 792–807. (10.1086/652244) 20687843

[45] Maina JN . 2022 Perspectives on the structure and function of the avian respiratory system: functional efficiency built on structural complexity. Front. Anim. Sci **3** , 851574. (10.3389/fanim.2022.851574)

[46] Duncker HR . 1974 Structure of the avian respiratory tract. Respir. Physiol. **22** , 1–19. (10.1016/0034-5687(74)90044-9) 4438848

[47] Macklem PT , Bouverot P , Scheid P . 1979 Measurement of the distensibility of the parabronchi in duck lungs. Respir. Physiol. **38** , 23–35. (10.1016/0034-5687(79)90004-5) 515560

[48] Watson RR , Fu Z , West JB . 2008 Minimal distensibility of pulmonary capillaries in avian lungs compared with mammalian lungs. Respir. Physiol. Neurobiol. **160** , 208–214. (10.1016/j.resp.2007.09.013) 17981521 PMC2692387

[49] West JB . 2009 Comparative physiology of the pulmonary blood–gas barrier: the unique avian solution. Am. J. Physiol. Regul. Integr. Comp. Physiol. **297** , R1625–R1634. (10.1152/ajpregu.00459.2009) 19793953 PMC2803621

[50] West JB , Fu Z , Deerinck TJ , Mackey MR , Obayashi JT , Ellisman MH . 2010 Structure–function studies of blood and air capillaries in chicken lung using 3D electron microscopy. Respir. Physiol. Neurobiol. **170** , 202–209. (10.1016/j.resp.2009.12.010) 20038456 PMC2821748

[51] Cozzi B , Bagnoli P , Acocella F , Costantino ML . 2005 Structure and biomechanical properties of the trachea of the striped dolphin Stenella coeruleoalba: evidence for evolutionary adaptations to diving. Anat. Rec. A **284** , 500–510. (10.1002/ar.a.20182) 15791584

[52] Davenport J , Cotter L , Rogan E , Kelliher D , Murphy C . 2013 Structure, material characteristics and function of the upper respiratory tract of the pygmy sperm whale. J. Exp. Biol. **216** , 4639–4646. (10.1242/jeb.083782) 24072789

[53] Davenport J , Jones TT , Work TM , Balazs GH . 2014 Unique characteristics of the trachea of the juvenile leatherback turtle facilitate feeding, diving and endothermy. J. Exp. Mar. Biol. Ecol. **450** , 40–46. (10.1016/j.jembe.2013.10.013)

[54] Moore C , Moore M , Trumble S , Niemeyer M , Lentell B , McLellan W , Costidis A , Fahlman A . 2014 A comparative analysis of marine mammal tracheas. J. Exp. Biol. **217** , 1154–1166. (10.1242/jeb.093146) 24311807

[55] Scheid P . 1979 Mechanisms of gas exchange in bird lungs. Rev. Physiol. Biochem. Pharmacol. **86** , 138–186. (10.1007/BFb0031533) 386468

[56] Barnas GM , Mather FB , Fedde MR . 1978 Response of avian intrapulmonary smooth muscle to changes in carbon dioxide concentration. Poult. Sci. **57** , 1400–1407. (10.3382/ps.0571400) 724602

[57] King AS , Cowie AF . 1969 The functional anatomy of the bronchial muscle of the bird. J. Anat. **105** , 323–336. 5802936 PMC1232137

[58] Wright AK , Ponganis KV , McDonald BI , Ponganis PJ . 2014 Heart rates of emperor penguins diving at sea: implications for oxygen store management. Mar. Ecol. Prog. Ser. **496** , 85–98. (10.3354/meps10592)

[59] Pütz K , Cherel Y . 2005 The diving behaviour of brooding king penguins (Aptenodytes patagonicus) from the Falkland Islands: variation in dive profiles and synchronous underwater swimming provide new insights into their foraging strategies. Mar. Biol. **147** , 281–290. (10.1007/s00227-005-1577-x)

[60] Watanuki Y , Kato A , Naito Y , Robertson G , Robinson S . 1997 Diving and foraging behaviour of Adelie penguins in areas with and without fast sea-ice. Polar Biol. **17** , 296–304. (10.1007/PL00013371)

[61] Wienecke B , Robertson G , Kirkwood R , Lawton K . 2007 Extreme dives by free-ranging emperor penguins. Polar Biol. **30** , 133–142. (10.1007/s00300-006-0168-8)

[62] Lanphier EH , Rahn H . 1963 Alveolar gas exchange during breath-hold diving. J. Appl. Physiol. **18** , 471–477. (10.1152/jappl.1963.18.3.471) 31083864

[63] Ridgway SH , Howard R . 1979 Dolphin lung collapse and intramuscular circulation during free diving: evidence from nitrogen washout. Science **206** , 1182–1183. (10.1126/science.505001) 505001

[64] Fahlman A , Moore MJ , Wells RS . 2021 How do marine mammals manage and usually avoid gas emboli formation and gas embolic pathology? Critical clues from studies of wild dolphins. Front. Mar. Sci. **8** , 25. (10.3389/fmars.2021.598633)

[65] Williams CL , Sato K , Shiomi K , Ponganis PJ . 2012 Muscle energy stores and stroke rates of emperor penguins: implications for muscle metabolism and dive performance. Physiol. Biochem. Zool. **85** , 120–133. (10.1086/664698) 22418705 PMC4887153

[66] Meir JU , Ponganis PJ . 2009 High-affinity hemoglobin and blood oxygen saturation in diving emperor penguins. J. Exp. Biol. **212** , 3330–3338. (10.1242/jeb.033761) 19801437

[67] Scheid P , Fedde MR , Piiper J . 1989 Gas exchange and air-sac composition in the unanaesthetized, spontaneously breathing goose. J. Exp. Biol. **142** , 373–385. (10.1242/jeb.142.1.373)

[68] Brackenbury JH , Gleeson M , Avery P . 1981 Effects of sustained running exercise on lung air-sac gas composition and respiratory pattern in domestic fowl. Comp. Biochem. Physiol. A **69** , 449–453. (10.1016/0300-9629(81)93003-6)

[69] Piiper J , Drees F , Scheid P . 1970 Gas exchange in the domestic fowl during spontaneous breathing and artificial ventilation. Respir. Physiol. **9** , 234–245. (10.1016/0034-5687(70)90073-3) 5445185

[70] Knower Stockard T , Heil J , Meir JU , Sato K , Ponganis KV , Ponganis PJ . 2005 Air sac P_O2_ and oxygen depletion during dives of emperor penguins. J. Exp. Biol. **208** , 2973–2980. (10.1242/jeb.01687) 16043602

[71] Williams CL , Czapanskiy MF , John JS , Leger JSt , Scadeng M , Ponganis PJ . 2021 Cervical air sac oxygen profiles in diving emperor penguins: parabronchial ventilation and the respiratory oxygen store. J. Exp. Biol. **224** , b230219. (10.1242/jeb.230219) 33257430

[72] Ponganis PJ , Stockard TK , Meir JU , Williams CL , Ponganis KV , Howard R . 2009 O_2_ store management in diving emperor penguins. J. Exp. Biol. **212** , 217–224. (10.1242/jeb.026096) 19112140 PMC2720999

[73] Ponganis PJ , Stockard TK , Meir JU , Williams CL , Ponganis KV , van Dam RP , Howard R . 2007 Returning on empty: extreme blood O_2_ depletion underlies dive capacity of emperor penguins. J. Exp. Biol. **210** , 4279–4285. (10.1242/jeb.011221) 18055617

[74] Boggs DF , Baudinette RV , Frappell PB , Butler PJ . 2001 The influence of locomotion on air-sac pressures in little penguins. J. Exp. Biol. **204** , 3581–3586. (10.1242/jeb.204.20.3581) 11707507

[75] Boggs DF . 1997 Coordinated control of respiratory pattern during locomotion in birds. Am. Zool. **37** , 41–53. (10.1093/icb/37.1.41)

[76] Scheid P , Piiper J . 1972 Cross-current gas exchange in avian lungs: effects of reversed parabronchial air flow in ducks. Respir. Physiol. **16** , 304–312. (10.1016/0034-5687(72)90060-6) 4644057

[77] Standiford TJ , Morganroth ML . 1989 High-frequency ventilation. Chest **96** , 1380–1389. (10.1378/chest.96.6.1380) 2510975

[78] Cieri RL , Farmer CG . 2016 Unidirectional pulmonary airflow in vertebrates: a review of structure, function, and evolution. J. Comp. Physiol. B **186** , 541–552. (10.1007/s00360-016-0983-3) 27062030

[79] Sato K , Shiomi K , Watanabe Y , Watanuki Y , Takahashi A , Ponganis PJ . 2010 Scaling of swim speed and stroke frequency in geometrically similar penguins: they swim optimally to minimize cost of transport. Proc. R. Soc. B **277** , 707–714. (10.1098/rspb.2009.1515) PMC2842743 19906666

[80] Ainley DG , Wilson RP . 2023 The aquatic world of penguins. Cham, Switzerland: Springer. (10.1007/978-3-031-33990-5)

[81] van Dam RP , Ponganis PJ , Ponganis KV , Levenson DH , Marshall G . 2002 Stroke frequencies of emperor penguins diving under sea ice. J. Exp. Biol. **205** , 3769–3774. (10.1242/jeb.205.24.3769) 12432000

[82] Smith RB , Sjöstrand UH . 1985 Apneic diffusion oxygenation and continuous flow apneic ventilation: a review. Acta Anaesthesiol. Scand. **29** , 101–105. (10.1111/j.1399-6576.1985.tb02167.x) 3883681

[83] Milsom WK , Jackson DC . 2011 Hibernation and gas exchange. Compr. Physiol. **1** , 397–420. (10.1002/cphy.c090018) 23737179

[84] Ponganis PJ , Williams CL , Kendall-Bar JM . 2024 Blood oxygen transport and depletion in diving emperor penguins. J. Exp. Biol **227** , jeb246832. (10.1242/jeb.246832) 38390686 PMC11006389

[85] Scott GR , Milsom WK . 2007 Control of breathing and adaptation to high altitude in the bar-headed goose. Am. J. Physiol. Regul. Integr. Comp. Physiol. **293** , R379–R391. (10.1152/ajpregu.00161.2007) 17491113

[86] Kawashiro T , Scheid P . 1975 Arterial blood gases in undisturbed resting birds: measurements in chicken and duck. Respir. Physiol. **23** , 337–342. (10.1016/0034-5687(75)90084-5) 238270

[87] Powell FL . 2000 Respiration. In Sturkie’s avian physiology (ed. GC Whittow ), pp. 233–264. San Diego, CA: Academic Press. (10.1016/B978-012747605-6/50011-0)

[88] Black CP , Tenney SM . 1980 Oxygen transport during progressive hypoxia in high-altitude and sea-level waterfowl. Respir. Physiol. **39** , 217–239. (10.1016/0034-5687(80)90046-8) 7375742

[89] Murrish DE . 1982 Acid-base balance in three species of Antarctic penguins exposed to thermal stress. Physiol. Zool. **55** , 137–143. (10.1086/physzool.55.2.30155848)

[90] Meir JU , Stockard TK , Williams CL , Ponganis KV , Ponganis PJ . 2008 Heart rate regulation and extreme bradycardia in diving emperor penguins. J. Exp. Biol. **211** , 1169–1179. (10.1242/jeb.013235) 18375841

[91] Powell FL , Hopkins SR . 2004 Comparative physiology of lung complexity: implications for gas exchange. Physiology **19** , 55–60. (10.1152/nips.01469.2003) 15016903

[92] Schmitt PM , Powell FL , Hopkins SR . 2002 Ventilation-perfusion inequality during normoxic and hypoxic exercise in the emu. J. Appl. Physiol. **93** , 1980–1986. (10.1152/japplphysiol.01108.2001) 12391060

[93] Williams CL , Meir JU , Ponganis PJ . 2011 What triggers the aerobic dive limit? Patterns of muscle oxygen depletion during dives of emperor penguins. J. Exp. Biol. **214** , 1802–1812. (10.1242/jeb.052233) 21562166 PMC3092726

[94] Butler PJ , Woakes AJ . 1984 Heart rate and aerobic metabolism in Humboldt penguins, Spheniscus humboldti, during voluntary dives. J. Exp. Biol. **108** , 419–428. (10.1242/jeb.108.1.419) 6423763

[95] Kooyman GL , Ponganis PJ . 1994 Emperor penguin oxygen consumption, heart rate and plasma lactate levels during graded swimming exercise. J. Exp. Biol. **195** , 199–209. (10.1242/jeb.195.1.199) 7964411

[96] Ponganis PJO , Williams C , Scadeng M . 2024 Supplementary material from: Respiratory anatomy and physiology in diving penguins. Figshare. (10.6084/m9.figshare.c.7599492) PMC11864836 40010382

